# Marathon race performance increases the amount of particulate matter deposited in the respiratory system of runners: an incentive for “*clean air marathon runs*”

**DOI:** 10.7717/peerj.11562

**Published:** 2021-06-17

**Authors:** Jerzy A. Zoladz, Zenon Nieckarz

**Affiliations:** 1 Department of Muscle Physiology, Institute of Basic Sciences, Faculty of Rehabilitation, University School of Physical Education, Kraków, Poland; 2 Experimental Computer Physics Department, Marian Smoluchowski Institute of Physics, Jagiellonian University, Kraków, Poland

**Keywords:** Air pollution, Health risk, Lung disease, Minute ventilation, Marathon running

## Abstract

**Background:**

In the last decades, marathon running has become a popular form of physical activity among people around the world. It should be noticed that the main marathon races are performed in large cities, where air quality varies considerably. It is well established that breathing polluted air results in a number of harmful effects to the human body. However, there have been no studies to show the impact of marathon run performance on the amount of the deposition of varied fractions of airborne particulate matter (PM) in the respiratory tract of runners. This is why the present study sought to determine the impact of marathon run performance in the air of varying quality on the deposition of the PM_1_, PM_2.5_, PM_10_ in the respiratory tract in humans.

**Methods:**

The PM_1_, PM_2.5_ and PM_10_ deposition was determined in an “average runner” (with marathon performance time 4 h: 30 min) and in an “elite marathon runner” (with marathon performance time 2 h: 00 min) at rest, and during a marathon race, based on own measurements of the PM content in the air and the size-resolved DF(*d*) profile concept.

**Results:**

We have shown that breathing air containing 50 µg m^−3^ PM_10_ (a borderline value according to the 2006 WHO standard - still valid) at minute ventilation (V_E_) equal to 8 L min^−1^ when at rest, resulted in PM_10_deposition rate of approximately 9 µg h^−1^, but a marathon run of an average marathon runner with the V_E_ = 62 L min^−1^ increased the deposition rate up to 45 µg h^−1^. In the elite runner, marathon run with the V_E_= 115 L min^−1^ increased PM_10_ deposition rate to 83 µg h^−1^. Interestingly, breathing the air containing 50 µg m^−3^of PM_10_ at the V_E_ = 115 L min^−1^by the elite marathon runner during the race resulted in the same PM_10_deposition rate as the breathing highly polluted air containing as much as 466 µg m^−3^ of PM_10_ when at rest. Furthermore, the total PM_10_ deposition in the respiratory tract during a marathon race in average runners is about 22% greater (203 / 166 = 1.22) than in elite runners. According to our calculations, the concentration of PM_10_in the air during a marathon race that would allow one not to exceed the PM_10_ deposition rate of 9 µg h^−1^should be lower than 10 µg m^−3^ in the case of an average runner, and it should be lower than 5.5 µg m^−3^ in the case of an elite runner.

**Conclusions:**

We conclude that a marathon run drastically increases the rate of deposition of the airborne PM in the respiratory tract of the runners, as a consequence of the huge V_E_ generated during the race. A decrease of the PM content in the air attenuates this rate. Based on our calculations, we postulate that the PM_10_ content in the air during a *“clean air marathon run”,* involving elite marathon runners, should be below 5.5 µg m^−3^.

## Introduction

Due to growing popularity of marathon running, the physiology/pathophysiology of marathon runners has attracted attention of several distinguished researches for over 70 years, as shown in the excellent topical issue of the Annals of the New York Academy of Science, edited by Milvy (1977). It should be underlined that since then the attention of scientists has been directed on the factors determining the level of marathon runners performance (Davies & Thompson, 1979; Sjödin & Svedenhag, 1985; Zoladz et al., 1993; Díaz, Fernández-Ozcorta & Santos-Concejero, 2018; Moir et al., 2019), with special focus in recent years on the physiological profile of runners who can break the barrier of 2 h in a marathon race (Skiba & Jones, 2011; Zoladz, Majerczak & Grassi, 2011; Elmer, Joyner & Carter, 2017; Hoogkamer, Kram & Arellano, 2017; Craig et al., 2019; Jones et al., 2020; Joyner et al., 2020).

In the last decades, marathon running over the classical distance of 42,195 m has also become a popular form of physical activity among people of varied age. It has been reported that nearly 1,300,000 people finished a marathon race in varied places around the world in the 2018, while the average marathon performance time reached ∼4 h: 30 min (Andersen, 2019). The current official world record in marathon running, established by Eliud Kipchoge (Kenya) is 2 h 1 min 39 s. It is worth mentioning that this runner recently completed the distance of marathon race in 1 h 59 min 40 s (unofficial record). This indicates that elite marathon runners are close to breaking the magic barrier of 2 h in an official marathon race (Jones et al., 2020; Joyner et al., 2020).

It should be underlined that major marathon races attracting close to 50,000 runners per race are performed in large, densely populated, cities such as New York, Boston, Chicago, London, Berlin or Tokyo (Carter, 2019). In vast urban areas of many cities around the world the levels of varied particulate matter (PM) concentrations in the air (Brook et al., 2010; Gupta et al., 2006; Huang et al., 2018; Huang et al., 2014) on a given day are far too high, compared to the current WHO air quality guidelines (WHO, 2006).

It is well documented that breathing polluted air with high PM concentration, even for a short time, increases the risk of several diseases in humans (Hoek et al., 2002; Brook et al., 2010; Du et al., 2016; Jo et al., 2017). It has been reported that more than 2 million premature deaths each year can be attributed to the effects of urban outdoor air pollution and indoor air pollution, and the health risk increases with level of air pollution (WHO, 2006, see also Table 1 therein).

It is has been shown that enhanced minute ventilation (V_E_) during varied physical activities – such as walking, jogging or cycling – increases the inflow of the PM_1_, PM_2.5_, PM_10_ and its deposition in the respiratory tract in humans (Daigle et al., 2003; Hussein et al., 2019). To our best knowledge, no study has been conducted so far that would show the impact of marathon running on the PM_1_, PM_2.5_, PM_10_ deposited in the respiratory tract of runners.

A growing number of scientific reports show that marathon running might cause serious, acute functional disturbances in the respiratory tract of runners, both during as well as just after a marathon run (for a review, see Tiller, 2019). This phenomena seems to be linked to the extraordinary stress, which the respiratory system undergoes during a marathon run. In order to maintain the required level of gas exchange during the race, a marathon runner has to increase the amount of the air ventilated per minute (V_E_), which can exceed 110 L per minute in highly trained athletes (for review see e.g., Hausswirth, Bigard & Guezennec, 1997). Such an increase of the V_E_ during a marathon race increases the inflow of airborne PM into the lungs and can increase the rate of its deposition in the respiratory tracts of runners.

This is why we attempted in the present study to determine the impact of marathon runs performed by average and by elite marathon runners on the amount of PM_1_, PM_2.5_, PM_10_ deposited in their respiratory tracks, when they perform their runs in normal air conditions (PM_10_ concentration below 50 µg m^−3^). Furthermore, we compared the level of PM deposition during the race to its level at rest. Finally, we assessed the impact of a marathon race on the daily PM deposition in the respiratory tract of marathon runners, when they run a marathon race in clean air.

## Material and Methods

### Minute ventilation

For the purpose of this study, we have assumed that the V_E_ in resting men, as well as in runners at rest would amount to 8 L min^−1^, while the V_E_ obtained by an average marathon runner (marathon performance time equal to 4 h 30 min (270 min)) during a marathon race amounts to 62 L min^−1^, i.e., ∼7–8 times higher than at rest. The V_E_ of the elite marathon runners (performance time 2 h (120 min)) during a marathon race was assumed to be 115 L min^−1^, i.e., ∼14–15 times higher than at rest. Our predictions of the V_E_ during a marathon race for an average runner and an elite runner are in agreement with experimental data collected in various groups of marathon runners, representing a broad range of performance levels (for details, see Maron et al., 1976; Mahler, Moritz & Loke, 1982; Hausswirth, Bigard & Guezennec, 1997).

### Air quality on the day of a marathon race

The measurements of the air quality were made in Kraków at the Jagiellonian University Campus by the University Measurement Station (UMS), (for details, see Nieckarz & Zoladz, 2020), located close to the green areas crossed by the marathon route (GPS location: 50.0291°N, 19.9046°E). In this paper, we used the data collected by this station on the day of an annual marathon in Kraków (“Cracovia Maraton”), i.e., on April 28th 2019. The station was equipped with digital laser dust sensor SEN0177 (DFRobot, China), which continuously measures the mass concentration of PM_1_ PM_2.5,_PM_10_ in the air and the concentration of suspended particulate matter (*C*) in the five size categories mentioned in Table 1. The data were used as a source of information to calculate total deposition by means of Eq. (1).

**Table 1:** Average total DF for five ranges of particle diameters, calculated for male exercise on the basis of the data collected in paper by Hussein et al. (2019).

Size range number	1	2	3	4	5
Diameter [µm]	0.3–0.5	0.5–1.0	1.0–2.5	2.5–5.0	5.0–10
Average total DF male-rest	0.135	0.220	0.558	0.865	0.884
Average total DF male-exercise	0.096	0.128	0.354	0.742	0.877

The sampling rate was 30 times per minute. Then, the data were aggregated to obtain average values per minute. Finally, we analysed the average values of concentrations that had been calculated on the basis of stored 1 min data. The station made measurements with the accuracy of 15% in a wide range of data (above 100 µg m^−3^) and inaccuracy equal ±10 µg m^−3^ below 100 µg m^−3^. The accuracy of the particulate matter detector used in our station in Kraków was verified by comparing the data recorded by the UMS station with the readings obtained from reference station EDM107 that belongs to the GRIMM company (Grimm & Eatough, 2009). According to the producer of the EDM107 analyser (the GRIMM company), the measurement error of this system is ± 2 µg m^−3^.

### Calculation of the total deposition

Total deposition (TD) of suspended PM in the human respiratory tract was calculated with Eq. (1), where: V is the volume of ventilated air [m^3^]; *n* is the number of a particle diameter range, DF_*n*_ is the average deposition fraction in *n*-th range; C_*n*_ is the concentration of PM in *n*-th range of diameter [m^−3^]; *ρ*_*n*_ – the aerosol effective density in *n*-th range [kg m^−3^] (see Table 2); *d*_*n*_  –the average particle diameter in the *n*-th range [m].

(1) \begin{eqnarray*}\mathrm{TD}=\sum _{n=1}^{5}\mathrm{V }\cdot {\mathrm{DF}}_{n}\cdot {\mathrm{C}}_{n}\cdot {\rho }_{n}\cdot \frac{\pi \cdot {d}_{n}^{3}}{6} \end{eqnarray*}

Deposition fraction (DF) can be calculated with Eq. (2), where: C_in_ and C_ex_ are, respectively, the inhaled and exhaled concentrations of suspended particulate matter; *d* is a particle diameter. The DF can also be calculated by applying average values of both concentrations for *n* ranges to Eq. (3), where C_ex,n_ and C_in,n_ are, respectively, the inhaled and exhaled concentrations of PM in *n*-th range.

(2) \begin{eqnarray*}\mathrm{DF} \left( d \right) =1- \frac{{\mathrm{C}}_{\mathrm{ex}} \left( d \right) }{{\mathrm{C}}_{\mathrm{in}} \left( d \right) } \end{eqnarray*}

(3) \begin{eqnarray*}{\mathrm{DF}}_{n}=1- \frac{{\mathrm{C}}_{\mathrm{ex},n}}{{\mathrm{C}}_{\mathrm{in},n}} \end{eqnarray*}

**Figure 1:**
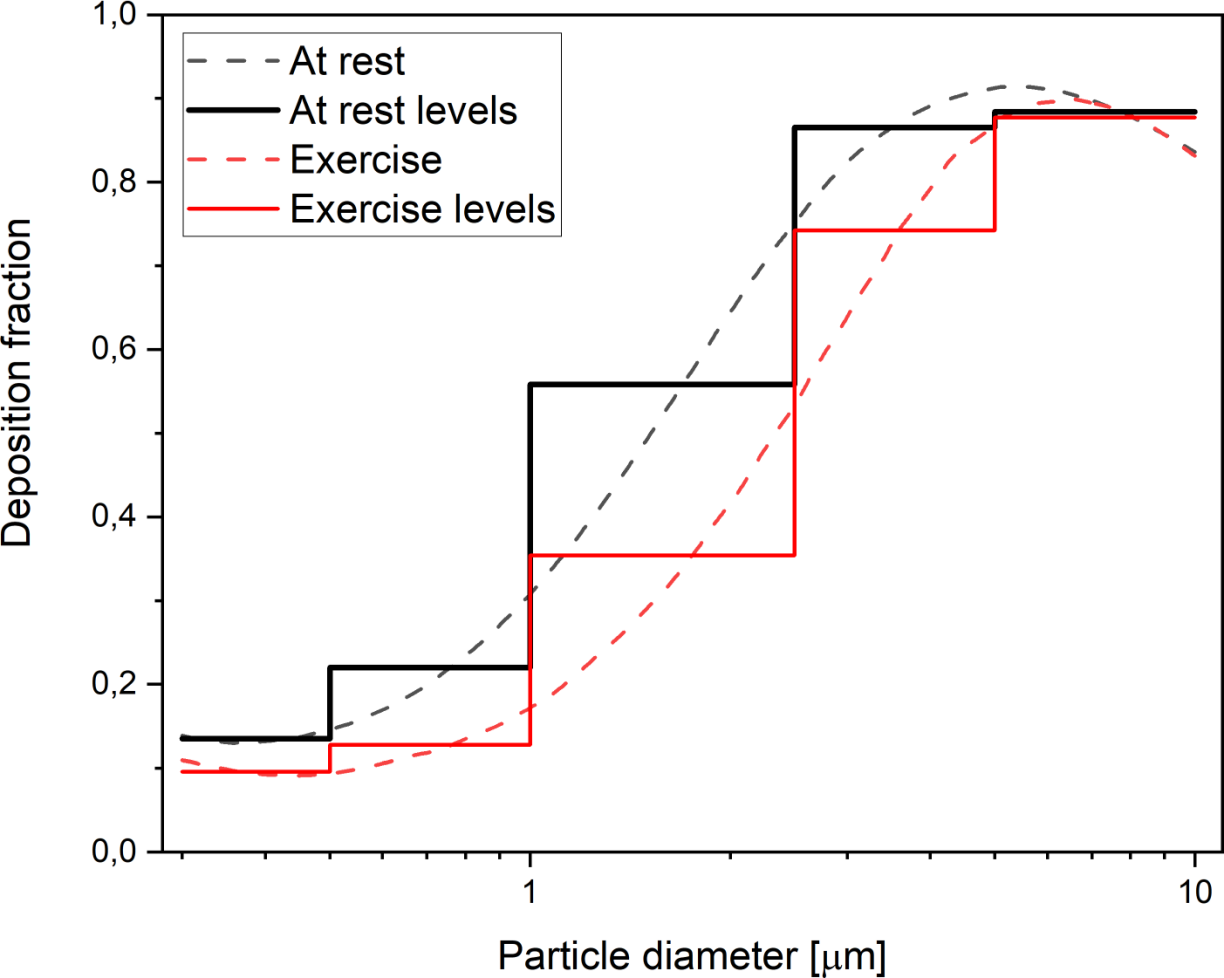
Size-resolved total DF in the respiratory tract in humans at rest and during exercise. Dashed curves present a continuous function, whereas solid levels present the average values of the five selected diameter rages, expressed in µm: [0.3–0.5), [0.5–1.0), [1.0–2.5), [2.5–5.0), [5.0–10.0), as proposed by Hussein et al. (2019). Colours: black–rest, red–exercise.

In this paper, size-resolved DF profiles proposed by Hussein et al. (2019) for a male at rest and a male during exercise have been used. The curves in Fig. 1 present–as a red line–a continuous function of size-resolved total DF in the respiratory tract of an adult for a male at rest (black line) and for a male during exercise (male-exercise) for the particle diameter range from 0.3 µm to 10 µm (Hussein et al., 2019).

**Table 2:** Urban aerosol effective densities according to the results obtained by Hu et al. (2012) and Wu & Boor (2020).

Size range number	1	2	3	4	5
Diameter [µm]	0.3–0.5	0.5–1.0	1.0–2.5	2.5–5.0	5.0–10
Effective particle densities *ρ* [kg/m^3^]	1,650	1,750	1,650	1,500	1,500

The black and red line levels in Fig. 1 present the average values calculated for the five selected diameter rages expressed in µm: [0.3–0.5), [0.5–1.0), [1.0–2.5), [2.5–5.0), [5.0–10.0), which correspond to the ranges implemented in the measurement station (UMS) to count suspended particulate matter. The values of D*F*_*n*_ for the five ranges are listed in Table 1.

## Results

### The quality of the air in Kraków on the day of the marathon race

**Figure 2:**
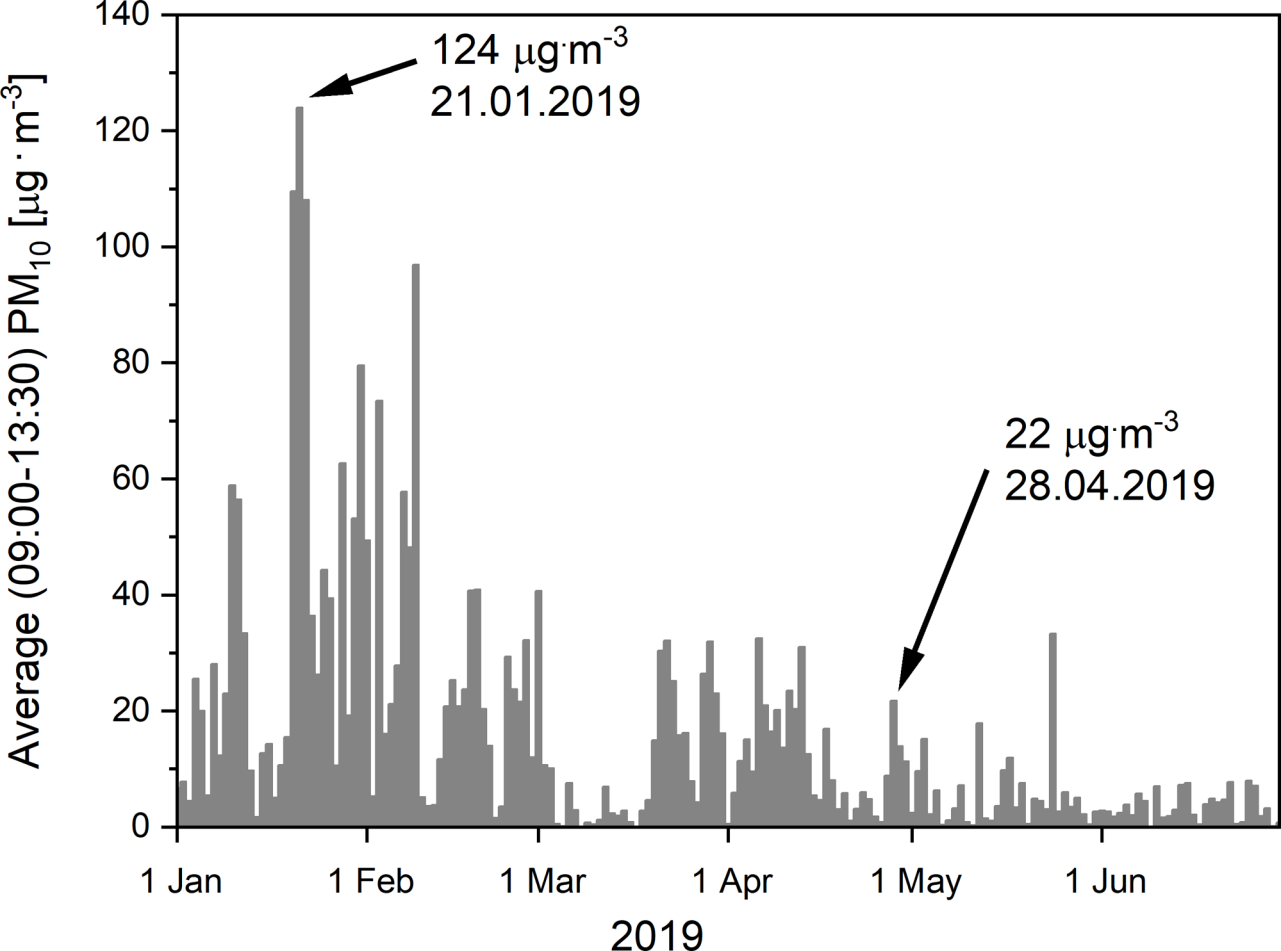
The PM_10_ concentrations in the air in Krakow between 1st January and 30th June, 2019. The level of the particulate matter concentration (PM_10_) in the air in Kraków, recorded in the morning hours, 9:00–13:30 (i.e., at the time of the marathon race under analysis, lasting 4 h: 30 min), in the period between 1st January and 30th June, 2019. Notice the moderate level of PM_10_ in the air that reached 22 µg m^−3^ on the 28th April, 2019 (the data of the 18th “Cracovia Maraton” race), as well as the peak level of PM_10_ recorded on the 21st January, 2019.

As presented in Fig. 2, the quality of the air in Kraków on the day of the marathon race (28th April, 2019), as judged on the basis of the amount of the PM_10_ in the air, fell within the 2006 WHO standard (still valid, see WHO, 2018), set at the level of 50 µg m^−3^ (WHO, 2006). It should be noticed that our measurements of the air quality in Kraków on the day of the marathon race showed a progressive decrease of PM_10_ in the air during the race. Its values reached 30 µg m^−3^, 23 µg m^−3^, 22 µg m^−3^ and 17 µg m^−3^ in the 1st, 2nd, 3rd and 4th hour of the marathon, respectively. This is why, we have used the average PM_10_ value amounting to 22 µg m^−3^ for some considerations made in this study. The observed progressive decline in the PM_10_ concentration during the race could be due to traffic restrictions imposed in the town for the 4 h of the race. Nevertheless, it should be noticed that, on some days in the early months of the year (January–February 2019), several times higher amounts of PM_10_ in the air were found in the morning / midday hours (9:00–13: 30), with the pick of 124 µg m^−3^ on 21st January, 2019. Moreover, it seems to be worth mentioning that on certain occasions in previous years (30 January 2017), even higher daily values of PM_10_ concentrations, reaching 696 µg m^−3^, have been recorded in the centre of Kraków (close the main traffic artery) (for a review, see Nieckarz & Zoladz, 2020).

**Figure 3:**
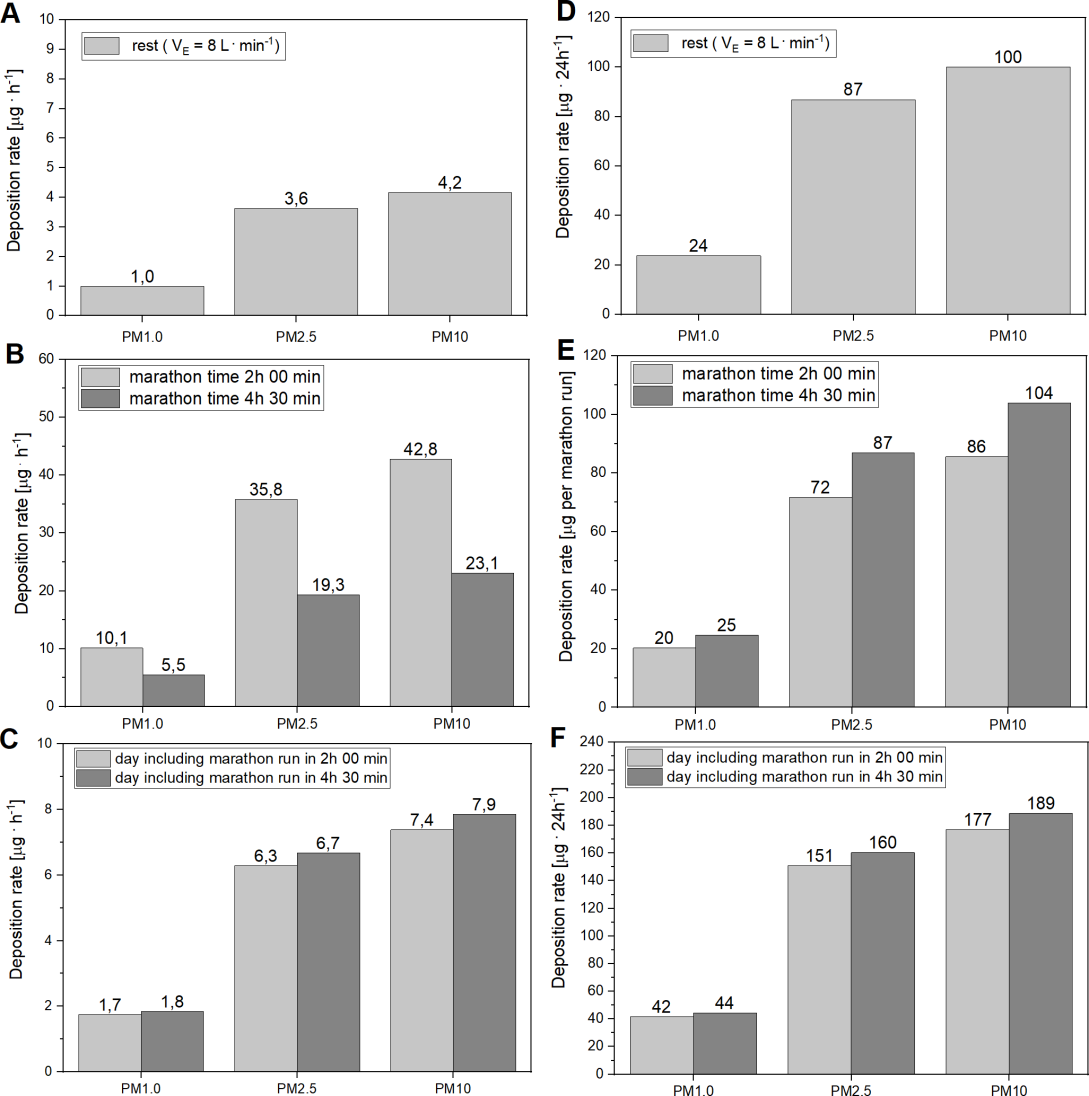
Deposition of PM_1_, PM_2.5_, PM_10_ in the respiratory tract in runners. (A and D) Deposition at rest. (B and E) During a marathon race. (C and F) Total daily deposition of PM in runners. (A and D) The values of the deposition rate at rest are expressed in [µg h^−1^] (A) and in [µg 24 h^−1^] (D) The calculations are based on the measurement of particulate matter concentrations (PM_1_, PM_2.5_, PM_10_) in the air in Kraków, as determined on the 28th April, 2019, as well as on the assumption that V_E_ is 8 L min^−1^ and on the profile of the total DF presented by Hussein et al. (2019). (B and E) The values of PM deposition rate during marathon race are expressed in [µg h^−1^] (B) and in [µg per marathon race] (E). The calculations of the PM_1_, PM_2.5_, PM_10_ depositions are based on the measurement of particulate matter concentration in the air in Kraków, as determined on 28th April, 2019, as well as on the assumption that the V_E_ during the marathon race in an elite marathon runner (marathon time 2 h: 00 min) was 115 L min^−1^, whereas in an average marathon runner (4 h: 30 min) the V_E_ was 62 L min^−1^. It was also assumed that the concentration of suspended particles in the air determined during the first 2 h of the race was constant up to the 4 h 30 min of the marathon race. Furthermore, for the purpose of calculations of the amount of varied fractions of PM deposited in the respiratory tract, we used the profile of the total DF for exercising males, as described by Hussein et al. (2019). (C and F) The values of the total daily deposition (during the marathon race plus the rest period) of the PM_1_, PM_2.5_, PM_10_ are presented for an elite runner (grey) and an average runner (black), expressed in [µg h^−1^] (C) and in [µg 24 h^−1^] (F). To calculate the amount of varied fractions of particulate matter deposited during the marathon run, it was assumed that the concentration of suspended particles in the air in Kraków, determined during the first 2 h of the race, was constant during the 4 h 30 min of the marathon race performed on 28th April, 2019. Furthermore, to calculate the level of deposition of varied fractions of PM during the race, we used the profile of the total DF for exercising males described by Hussein et al. (2019), whereas to calculate the level of PM deposition at rest, we applied the profile of the total DF for male at rest, as described by Hussein et al. (2019).

### Deposition of suspended particulate matter at rest

Figures 3A and 3B presents levels of deposition of PM_1_, PM_2.5_, PM_10_ in the respiratory tract of runners at rest (both average and elite runners, assuming that their V_E_ at rest is 8 L min^−1^), expressed in µg h^−1^ (Fig. 3A) and in µg 24 h^−1^ (Fig. 3B).

### Deposition of suspended particulate matter during a marathon race

Figures 3B and 3E present the values of deposition of PM_1_, PM_2.5_, PM_10_ in the respiratory tract in an elite and an average marathon runner during the race, expressed in µg h^−1^ (Fig. 3B) and in µg per marathon race (Fig. 3E).

### Daily deposition of suspended particulate matter on the day of the marathon race

We also calculated the total deposition of varied fractions of PM_1_, PM_2.5_, PM_10_ in the respiratory tract in elite and in average runners on the day (24 h) of the marathon, while taking into consideration their deposition during the race and in the remaining time of the 24 h period, when runners were at rest (see Figs. 3C and 3F).

### Impact of air quality on PM deposition in the respiratory tract during marathon race

**Figure 4:**
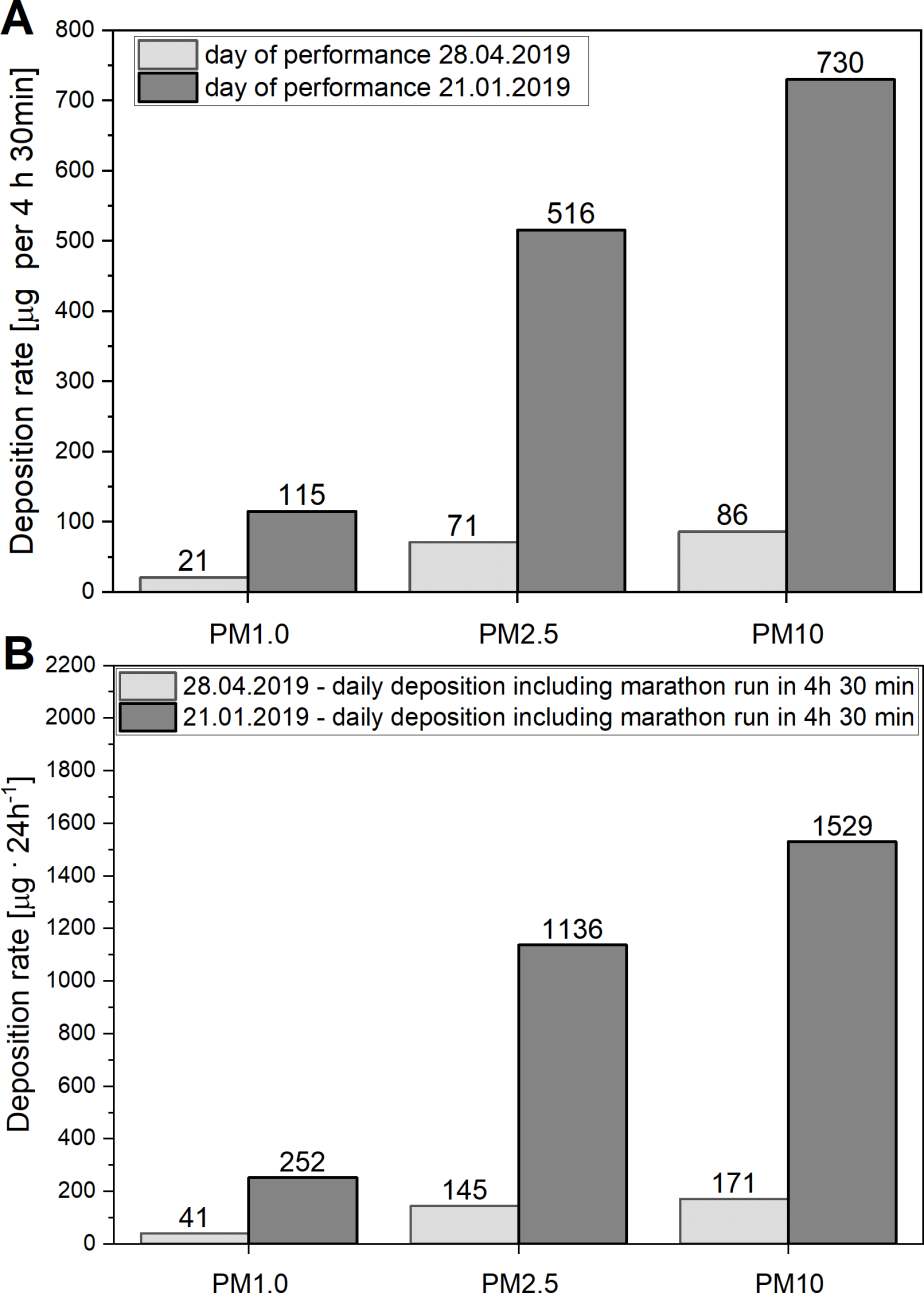
(A) Deposition of particulate matter in the respiratory tract in runners during marathon races. (B) Levels of total daily deposition of varied fractions of PM in runners. (A) Levels of deposition of varied fractions of particulate matter (PM_1_, PM_2.5_, PM_10_) in the respiratory tract of an average runner during two marathon races, calculated with the data of the air quality (PM_10_ = 22 µg m^−3^) register between 9:00–13:30 on 28th April, 2019 (the day of the 18th “Cracovia Maraton”) and on 21st January, 2020 that witnessed the worst quality of the air in Krakow (PM_10_ = 124 µg m^−3^), (in the period between 1st January and 30st Jun, 2019) (see also Fig. 2). (B) Total daily deposition of varied fractions of particulate matter (PM_1_, PM_2.5_, PM_10_) in the respiratory tract (during the marathon race plus the period of rest) in an average runner, calculated with the data of the air quality (PM_10_ = 22 µg m^−3^), register between 9:00–13:30 on 28th April 2019 (the day of the 18th “Cracovia Maraton”) and on day of 21st January 2019 that witnessed the poorest quality of the air in Krakow (PM_10_ = 124 µg m^−3^) (in the period between 1st January and 30st June 2019) (see also Fig. 1).

Figure 4A prensents the results of calculations of depositions of the PM_1_, PM_2.5_, PM_10_ in the respiratory tract of runners during two marthon races: the first one performed in relatively clean air (PM_10_ = 22 µg m^−3^) on 28th April 2020, and the second one pefformed in more poluted air (PM_10_ = 124 µg m^−3^), as recorded in Kraków on 21st Januray, 2020 (see Fig. 2). Note huge diffrences between these two races in the deposition rates applicable to an average runner.

### Impact of air quality on daily deposition of PM in the respiratory tract in runners

As presented in Fig. 4B, the increase of air pollution showed by comparing the measurements performed on 21st January and on 28th April 2020 (see Fig. 2) had a strong impact on the daily deposition of PM_1_, PM_2.5_, PM_10_ in the respiratory tract in runners. It can be seen that the levels of deposition of varied fractions of PM on 21st January 2020 are about 5.5−8.5 times greater than on the 28th April 2020 (see Fig. 4B).

The calculations of the level of deposition of varied fractions of particulate matter at rest and during a marathon run are based on real concentrations of suspended particles in the air registered during the 4 h 30 min of the marathon race and during the remaining 19 h 30 min of rest.

**Figure 5:**
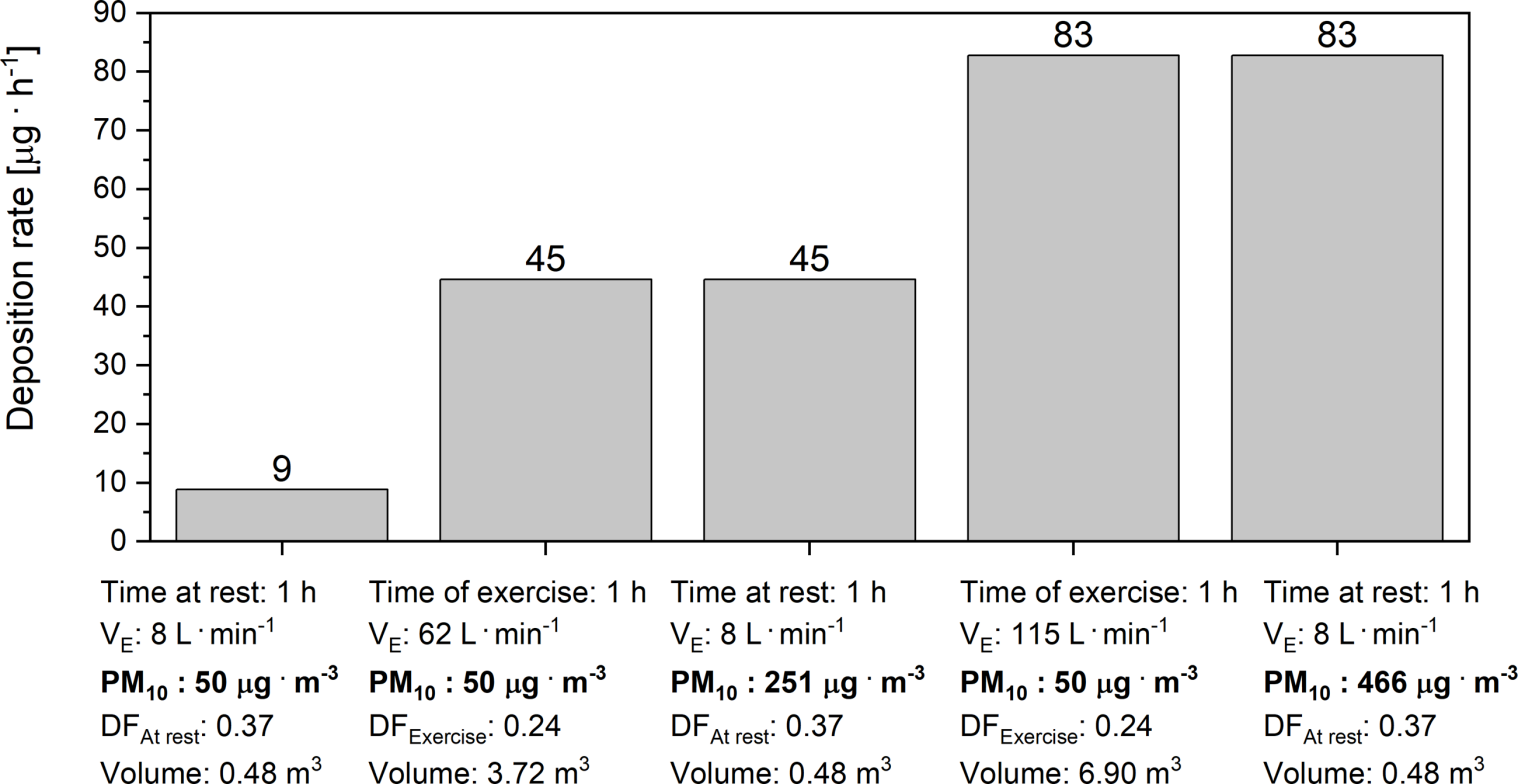
Deposition rate of PM_10_ in the respiratory tract at rest and during a marathon run. Levels of deposition rate of PM_10_ in the respiratory tract at rest and during a marathon race, performed by an average runner (4 h: 30 min) as well as by an elite marathon runner (2 h: 00 min). Notice also the levels of simulated PM_10_ content in the air, required to increase the rate of PM_10_ deposition in the respiratory tract of runners at rest to the level witnessed during marathon races (50 µg m^−3^ vs. 251 µg m^−3^ vs. 466 µg m^−3^). Where: V_E_ –minute ventilation; Volume - the volume of the air filtered by respiratory tract during the time of exercise, PM_10_ –the mass concentration of particles with an aerodynamic diameter smaller than 10 µm, DF_At rest_ –the average mass deposition fraction of PM_10_ for a male at rest, DF_Exercise_ –the average mass deposition fraction of PM_10_ for an exercising male.

### Comparison of the rate of PM_10_ deposition in the respiratory tract in runners at rest and during a marathon race performed in the air with the PM_10_= 50 µg m^−3^

As presented in Fig. 5, the increase of minute ventilation from its level at rest up to 62 L min^−1^ in an average runner increased the rate of PM_10_ deposition in the respiratory tract of runners from 9 to 45 µg h^−1^. In the case of elite runners, the increase of the V_E_ from its level at rest (8 L min^−1^) up to 115 L min^−1^ during the marathon race, performed within 2 h: 00 min, resulted in an increase of the rate of PM_10_ deposition in the respiratory tract from 9 up to 446 µg h^−1^. As shown in Fig. 5, we estimated that performance of a marathon race by an average marathon runner breathing in the air with PM_10_ = 50 µg m^−3^ at V_E_ = 62 L min^−1^ will result in the same rate of the PM_10_ deposition as in the case of breathing the air with PM_10_ = 251 µg m^−3^ when at rest (V_E_ = 8 L min^−1^). We have also calculated that performance of a marathon race by an elite marathon runner (2 h: 00 min) breathing in the air with PM_10_ = 50 µg m^−3^ at V_E_ = 115 L min^−1^ during the race will result in the same rate of the PM_10_ deposition in the respiratory tract as in the case of breathing the air with PM_10_ = 446 µg m^−3^ when at rest (V_E_ = 8 L min^−1^). The values of the factors used in these calculations (DF_At rest_ and DF_Exercise_), namely 0.37 and 0.24, respectively, represent the mass deposition fraction factors for PM_10_ calculated on the basis of real data, presented and analysed earlier (see (4)** and Fig. 5). These values are in agreement with the results of previous studies (Guo et al., 2020; Rissler et al., 2017).

(4) \begin{eqnarray*}D{F}_{At rest/Exercise}= \frac{\mathrm{TD}}{\mathrm{M}} \end{eqnarray*}

Where: TD - total deposion of PM_10_ calculated with Eq. (1) (using DF for male-rest and male-exercise, respectively, according to Table 1) in the volume of ventilated air (V), M - is the mass of PM_10_ in the same volume V.

**Figure 6:**
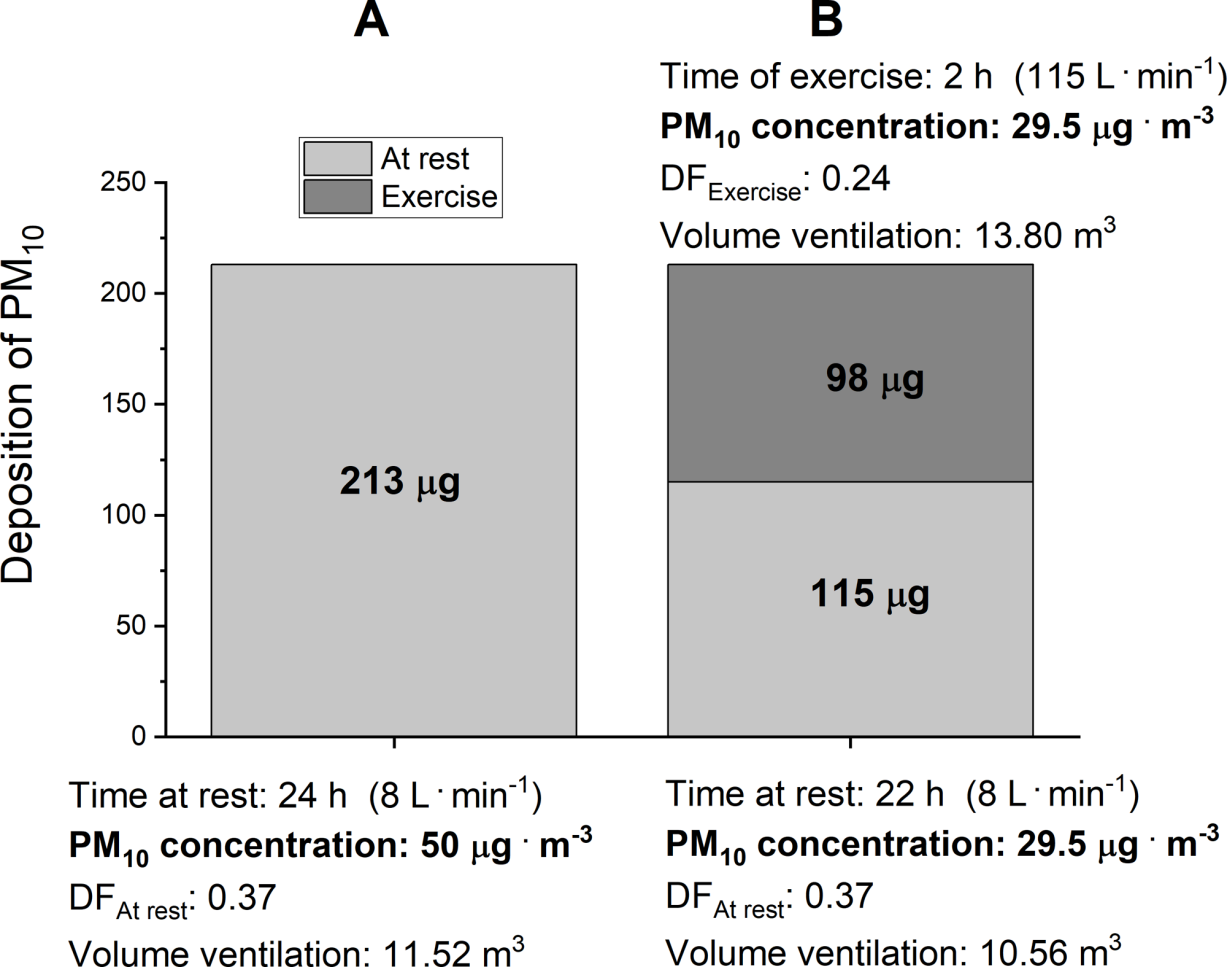
Daily deposition of PM_10_ when breathing in the air with PM_10_= 50 ug m^−3^. (A) presents the levels of daily (24 h) deposition of PM_10_ at rest when breathing in the air with PM_10_ = 50 ug m^−3^ at V_E_ = 8 L min^−1^ (B) presents the quality of the air required to obtain the same deposition of PM_10_ during the day of a marathon race (rest plus marathon race performed in 2 h: 00 min) as on the day when breathing the air with PM_10_ = 50 ug m^−3^ at V_E_ = 8 L min^−1^ when at rest. Where: V_E_ –minute ventilation; Volume –the volume of the air filtered by the respiratory tract during the time of exercise, PM_10_ –the mass concentration of particles with an aerodynamic diameter smaller than 10 µm, DF_At rest_ –the average mass deposition fraction of PM_10_ for a male at rest, DF_Exercise_ –the average mass deposition fraction of PM_10_ for an exercising male.

We also calculated the daily (24 h) amount of the PM_10_ deposition in the respiratory tract in a runner at rest, while breathing the air containing 50 µg m^−3^ of PM_10_, with the V_E_ of 8 L min^−1^. In these conditions, the daily PM_10_ deposition reached 213 µg per 24 h (see Fig. 6A). As presented in Fig. 6B, in order not to exceed the basal level of daily PM_10_ deposition of 213 µg per 24 h, the air on the day when an elite runner runs in a marathon race should contain no more than 29.5 µg m^−3^ of PM_10_ (see Fig. 6).

**Figure 7:**
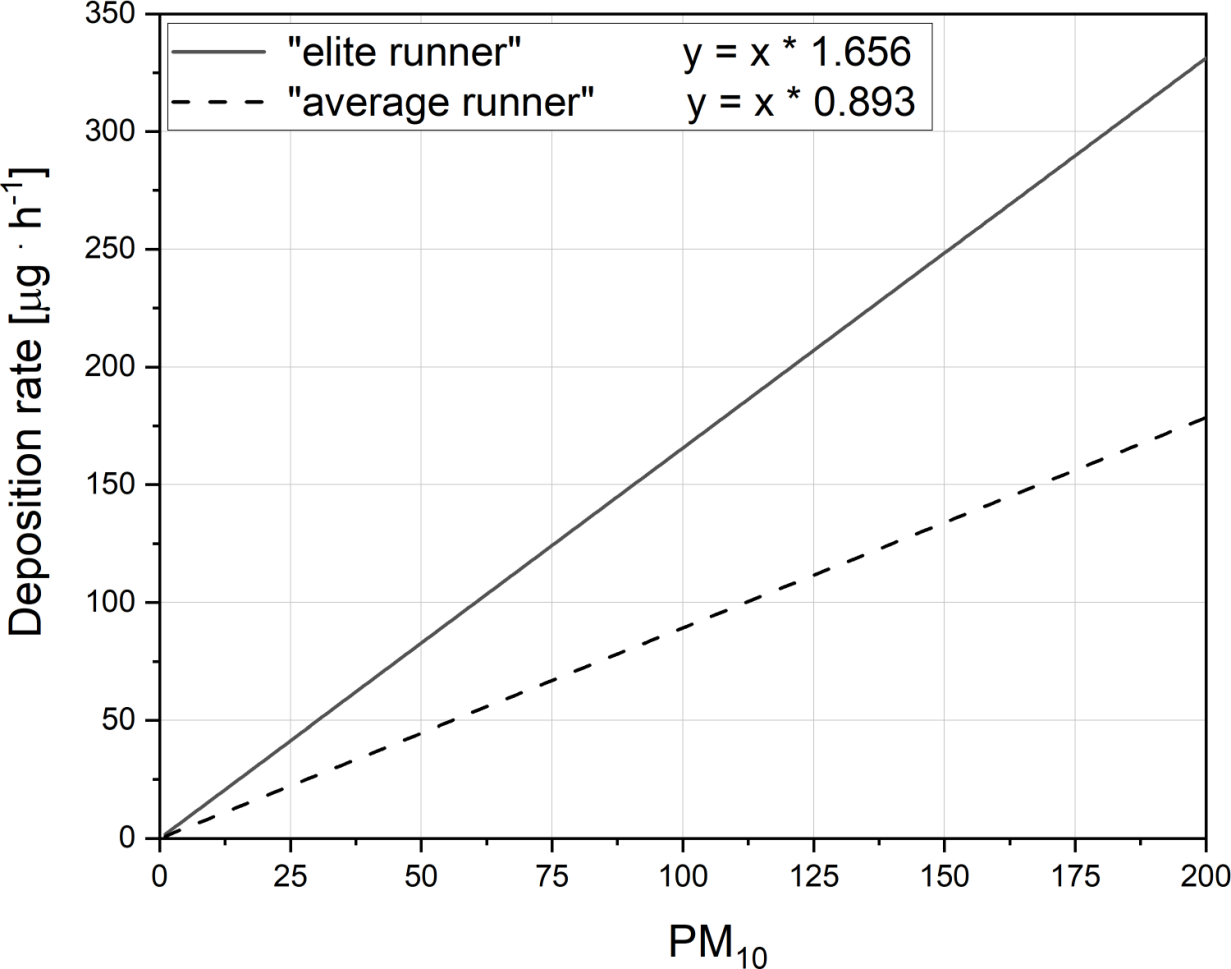
The relationship between PM_10_ content in the air its deposition rate during the marathon race. Figure presents the impact of varied PM_10_ content (µg m^−3^) in the air on the deposition rate of PM_10_ in the respiratory tract (µg h^−1^) during a marathon race, performed by an average runner (4 h: 30 min) (dashed line) as well as by an elite marathon runner (2 h: 00 min) (solid line). Notice also the 2 times greater slope in this relationship in case of an elite runner when compared the average runner.

We have also evaluated the relationship between PM_10_ content in the air and its deposition rate during the marathon race performed by an average runner as well as by an elite marathon runner (Fig. 7). As presented in Fig. 7 the deposition rate of PM_10_ in the respiratory tract of the runners during a marathon race is linearly dependent upon the PM_10_ content in the air. The ∼2 times greater slope in this relationship in case of an elite runner when compared the average runner (Fig. 7), indicates that a given PM_10_ content in the air the deposition rate of PM_10_ in the elite runner is greater than in the average runner.

## Discussion

It is well documented that a classic marathon run performed over the distance of 42 195 m exerts serious demands on various systems of the human body (for an overview, see e.g., Pedersen et al., 2001; Elmer, Joyner & Carter, 2017; Hagan 3rd, 2018; Joyner et al., 2020). In order to extend the knowledge concerning potential health-risks associated with marathon performance, this paper presents new data showing the impact of marathon race performance on the amount of airborne particulate matter deposited in the respiratory tract in runners. This issue seems to be very relevant in the present time, since the main marathon races are performed in large cities (Andersen, 2019), where the air quality varies considerably (see, for instance*,* Monforti-Ferrario et al., 2018; Dantas et al., 2020; Lal et al., 2020; Rattigan et al., 2020).

### PM deposition rate at rest

As presented in Figs. 3A and 3D, PM deposition rates, assessed in our study in humans at rest on 28th April 2019 (when PM_10_ concentration in the air was 22 µg m^−3^) reached about 1, 3.63 and 4.17 µg h^−1^ (Figs. 3A) and 24, 87 and 100 µg 24 h^−1^ (Figs. 3D), for PM_1_, PM_2.5_ and PM_10_, respectively.

### Impact of running a marathon race in “clean air” on PM deposition in humans

In the present study, we have demonstrated that a marathon race performed even in relatively clean air containing only 22 µg m^−3^ of PM_10_ dramatically increased the rate of deposition of PM_1_, PM_2.5_ and PM_10_ in the respiratory tract of marathon runners during the race. So that performance of a marathon race resulted in increased rates of PM deposition in the respiratory tract of average runners, reaching up to about 5.56, 19.34, and 23.12 µg h^−1^ for PM_1_, PM_2.5_ and PM_10_, respectively (Fig. 3B). In the case of an elite marathon runner, this PM deposition rate (expressed in µg h^−1^) was about two times higher than in the case of an average runner and reached 10, 36 and 43.0 µg h^−1^ for PM_1_, PM_2.5_ and PM_10_, respectively (Fig. 3B).

Interestingly, the deposition rate of varied PM – expressed in µg per marathon race for all studied fractions (PM_1_, PM_2.5_ and PM_10_) – was systematically higher in average runners than in the elite (see Fig. 3E). It shows that a marathon race, in the case of elite runners, generates a higher pollution stress for the respiratory system expressed in µg of PM per unit of time than in the case of average runners, but the total amount of the PM deposited during the race is clearly greater in average runners than in elite marathon runners (see, Fig. 3E). The main reason for a greater rate of PM deposition rate expressed in [µg h^−1^] found in elite runners is higher V_E_ during a marathon race (115 L min^−1^ vs. 62 L min^−1^ in elite and in average runners, respectively). In turn, greater PM deposition during a marathon run expressed in [µg h^−1^] that was observed in average runners (as shown in Fig. 3E) is caused by greater total V_E_ needed for average runners to cover the marathon distance (see also Figs. 6 and 7). The required higher total V_E_ during a marathon race in an average runner is due to two factors: (i) poorer running economy (i.e., higher oxygen cost of running) and (ii) higher body mass, when compared to elite runners (for overview see e.g., Maron et al., 1976; Mahler, Moritz & Loke, 1982; Hausswirth, Bigard & Guezennec, 1997; Elmer, Joyner & Carter, 2017; Hoogkamer, Kram & Arellano, 2017; Joyner et al., 2020).

### Impact of marathon run in varied air quality on PM deposition in humans

As shown in Figs. 3C and 3D, running the marathon distance in Kraków on the day of 28th April 2019 (see Fig. 2) almost doubled the amount of PM_1_, PM_2.5_ and PM_10_ deposited in the respiratory tract of marathon runners per 24 h, when compared with a day spend at rest in the same air quality (see comparisons in Figs. 3A and 3C). This indicates that marathon race performance even in relatively clean air strongly influences the amount of PM_1_, PM_2.5_ and PM_10_ deposited in the respiratory tract of humans.

As mentioned above, so far we have discussed the levels of the deposition of PM_1_, PM_2.5_ and PM_10_ in the respiratory tract of runners who exercised in relatively clean air (containing “only” 22 µg m^−3^ of PM_10_), according to measurements performed in Kraków on 28th April 2019 (see Fig. 2). It should be noticed, however, that the air quality in Kraków on some days can be much poorer than that on the day of performance of the 18th “Cracovia Maraton” race. For example, the daily PM_10_ concentration in the air in Krakow on 21st January 2019 reached 124 µg m^−3^. It was found that the levels of deposition of PM_1_, PM_2.5_, PM_10_ in the respiratory tract during the marathon race in an average runner who exercised on 21th January 2019 reached up to 115 µg, 516 µg, 730 µg, respectively, whereas on 28th April 2019 the values were 21 µg, 71 µg, 86 µg, respectively (see Fig. 4A). We also aimed at answering the question concerning the effect of running the marathon distance by elite runners in more polluted air (PM_10_ content = 124 µg m^−3^) on PM deposition in their respiratory track. It was found out that the levels of deposition of PM_1_, PM_2.5_ and PM_10_ in their respiratory tract during the marathon race on 21th January 2019 would reach 113 µg, 511 µg, 726 µg, respectively, whereas on of 28th April 2019, the values were 20 µg, 72 µg, 86 µg, respectively (see Fig. 3B). Furthermore, in the case of an elite marathon runner participating in the marathon race on 21th January 2019, with the V_E_ of 115 L min^−1^ during the race, PM_10_ deposition rate was 206 µg h^−1^, i.e., 411 µg of PM_10_ per marathon race. It implies that an elite marathon runner breathing the air containing 124 µg m^−3^ of PM_10_ at the V_E_ = 115 L min^−1^ during a race would experience the same PM_10_ deposition rate as in the case of breathing very highly polluted air –containing as much as 1 156 µg m^−3^ of PM_10_ –when at rest. Accordingly, the daily amounts of varied PM deposited on 21st January 2019 would be several times greater than on 28th April 2019. The situation is even more dramatic in the case of the worst daily air quality levels measured by us on 30th January 2017. Namely, the PM_10_ concentration in the air in Kraków that reached 696 µg m^−3^ on that day (which is not exceptional for large cities; see, for instance, Mage et al., 1996; Marlier et al., 2016), in the case of an elite athlete who covers the marathon distance within 2 h 00 min, would result in an extremely high deposition rate of PM_10_, reaching up to to 1,152 µg h^−1^, i.e., 2,305 µg of the PM_10_ per marathon race. According to this scenario, an elite marathon runner breathing the air containing up to 696 µg m^−3^ of PM_10_ at the V_E_ = 115 L min^−1^ during the race would experience the same PM_10_ deposition rate as a runner breathing extremely polluted air–containing as much as 6 490 µg m^−3^ of PM_10_–when at rest. So poor air quality–when the concentration of PM_10_ in the air is at the level of 6.50 mg m^−3^–exceeds the levels of PM_10_ concentration measured in the most polluted parts of coal mines (Kizil & Donoghue, 2002).

### Impact of marathon run performed in the air containing up to 50 µg m^−3^ of PM_10_

According to WHO air quality guidelines (WHO, 2006), the upper limit for the content of PM_10_ in the air is 50 µg m^−3^. For this reason, we assessed the rate of PM_10_ deposition in the respiratory tract in humans at rest, during a marathon run by an average runner as well as during a marathon run by an elite athlete, while breathing the air containing up to 50 µg m^−3^ of PM_10_. As shown in Fig. 5, breathing the air at (V_E_) equal to 8 L min^−1^ when at rest resulted in the PM_10_ deposition rate of ∼9 µg h^−1^, but a marathon run by an average marathon runner with V_E_ = 62 L min^−1^ increased the deposition rate up to 45 µg h^−1^. In an elite runner, a marathon run at the V_E_ = 115 L min^−1^ increased the PM_10_ deposition rate up to 83 µg h^−1^. Interestingly, if an elite marathon runner breathed the air containing 50 µg m^−3^ of the PM_10_ at the V_E_ = 115 L min^−1^ during the race, it would result in the same PM_10_ deposition rate as in the case of breathing very polluted air–containing as much as 466 µg m^−3^ of PM_10_–when at rest. This is why, in order to minimize this health risk, runners should avoid running marathon races in cities, where the air is highly polluted. We also calculated that, in order to not exceed the daily PM deposition in the respiratory tract due to breathing the air containing 50 µg m^−3^ of PM_10_ at rest, the PM_10_ concentration in the air on the day of a marathon race should not exceed 29.5 µg m^−3^ (see Fig. 6B). In the subsequent consideration we went a step further and calculated the maximum PM_10_ concentration in the air on the day of a marathon run that would be required for this level of PM_10_ deposition rate not to be exceeded in runners participating in a marathon race. According to our calculations, the concentration of PM_10_ in the air during a marathon race that would allow one not to exceed the PM_10_ deposition rate of 9 µg h^−1^ should be lower than 10 µg m^−3^ in the case of an average runner, and it should be lower than 5.5 µg m^−3^ in the case of an elite runner. This seems to be the upper limit of PM_10_ in the air for marathon runners on the day of a marathon race in a given city. Interestingly, it has also been reported that even very moderate air pollution can negatively affect marathon performance in women (Marr & Ely, 2010). The paper reports that women’s marathon performance was slower by 1.4% for every 10 µg m^−3^ of increase in PM_10_ concentration in the air (in range of 4.5–42 µg m^−3^ of PM_10_–see Figure 1 in Marr & Ely (2010).

### Health risk related to marathon running

It is well documented that marathon running often leads to serious health problems and, in extreme situations, it ends with runner’s death (see, for instance Cohen & Ellis, 2012; Kim et al., 2012; Siegel & Noakes, 2017; Dayer & Green, 2019). It was estimated that the risk of death during marathon race is approximately 1 death per 149,968 participants, being more than twice higher in males (one per 102,503) than in females (one per 243,879) (Dayer & Green, 2019). Poor health status of (some) marathon participants one of the factors that strongly increases the risk of health complications and deaths during a marathon race (Tsiflikas et al., 2015; Herm et al., 2017; Churchill et al., 2020). Strenuous endurance training– even when performed in laboratory conditions– leads to several adaptive responses in lungs tissue mitochondria (Jarmuszkiewicz et al., 2020). This indicates that physical exercise is indeed very challenging to the lungs when performed even in clear air. Air pollution, however, is another factor, which–in our opinion–should receive more attention as a potential health threat to marathon runners. The main reason for this is the fact that running a marathon race enhances minute ventilation by about 7–8 times in the case of an average runner and by about 15 times in the case of an elite runner. This huge increase of V_E_ during exercise, required to maintain muscle cell energy homeostasis during strenuous exercise (for overview see Zoladz et al., 2013; Zoladz et al., 2014; Grassi, Rossiter & Zoladz, 2015; Zoladz, Grassi & Szkutnik, 2019), increases the inflow of varied PM into the respiratory tract of runners and enhances its deposition therein (see Fig. 7). This leads to an increased risk of varied diseases (see, for instance Hoek et al., 2002; Brook et al., 2010; Du et al., 2016; Jo et al., 2017), including reported acute lungs dysfunctions in marathon runners (see Tiller, 2019). Taking in to account the fact that the air quality in several large cities that organize marathon runs can be questionable (see, for instance Monforti-Ferrario et al., 2018; Dantas et al., 2020; Lal et al., 2020; Rattigan et al., 2020), runners should pay more attention to this fact when planning to participate in marathon races (see also Morici et al., 2020).

According to the considerations we have presented above, in order to minimalize the health risk of the marathon runs, we postulate introducing the concept of the “*clean air marathon runs”.* Based on our calculations we predict that the PM_10_ content in the air during *“clean air marathon run”* should stay below 10 µg m^−3^ in the case of an average runner and it should be lower than 5.5 µg m^−3^ in the case of an elite runner. We propose that only the marathon races which fulfil this standard could constitute a prestige league of the *“clean air marathon runs”.*

It should be mentioned that recent studies particularly underline negative impact of ultrafine particles (UFP, diameter below 0.1 µm) in the air on various body organs (see e.g., Bevan et al., 2020; Daiber et al., 2020; Niu et al., 2020; Shkirkova et al., 2020). Unfortunately, continuous field measurements of the UFP level in the air are conducted very rarely. At this time, within the framework of our study, we were also unable to perform such measurements. This is why further studies are needed to show the impact of acute exposure to high levels of UFP (e.g., during performance of a marathon race in polluted air) on their deposition in the lungs and on the health of humans.

Summing up: our study showed that running the marathon distance even in relatively clean air drastically increases (from five to nine times) the rate of deposition of varied airborne PM in the respiratory tract of runners, compared to its level at rest. This rate during a marathon race is about 100% greater in elite runners than in average runners (see Fig. 7), but the total PM_10_ deposition in the respiratory tract during a marathon race in average runners is by about 22% greater (203 / 166 =1.22) than in elite runners. This huge rate of deposition of varied airborne PM in the respiratory tract of runners caused by marathon runs requires more attention, in order to develop a strategy aimed at decreasing the health risk to runners. The simplest yet effective approach aimed at minimizing the level of deposition of varied airborne PM in the respiratory tract of runners consists in avoiding marathon runs in cities, where the air is highly polluted.

## Conclusions

A marathon run drastically increases the rate of deposition of the airborne PM in the respiratory tract of the runners, as a consequence of the huge V_E_ during the race. A decrease of the PM content in the air attenuates this rate. Based on our calculations, we postulate that the PM_10_ content in the air during a *“clean air marathon run”,* involving elite marathon runners, should be below 5.5 µg m^−3^.

##  Supplemental Information

10.7717/peerj.11562/supp-1 Supplemental Information 1 Row data for Fig. 2

## Additional Information and Declarations

### Data Availability

The following information was supplied regarding data availability: The raw measurements presented in Fig. 2 are available in the Supplemental Files.
